# *MC1R* variants predisposing to concomitant primary cutaneous melanoma in a monozygotic twin pair

**DOI:** 10.1186/1471-2350-13-81

**Published:** 2012-09-14

**Authors:** Cristina Pellegrini, Maria Concetta Fargnoli, Mariano Suppa, Ketty Peris

**Affiliations:** 1 Department of Dermatology, University of L’Aquila, L’Aquila, Italy; 2 Department of Dermatology, University of L’Aquila, via Vetoio - Coppito 2, 67100, L’Aquila, Italy

**Keywords:** Melanoma, Monozygotic twins, *CDKN2A*, *CDK4*, *MC1R*, *MITF*, Genetic susceptibility

## Abstract

**Background:**

Concomitant primary cutaneous melanoma in monozygotic twins has been reported in only two pairs but in neither of them genetic analysis was performed. Two high-penetrance susceptibility genes, *CDKN2A* and *CDK4* and one low-penetrance gene, *MC1R,* are well-defined genetic risk factors for melanoma. *MITF* has been recently identified as a novel intermediate risk melanoma-predisposing gene.

**Case presentation:**

We describe the extraordinary occurrence of a primary cutaneous invasive melanoma in two 44-year-old identical, female twins, on the same body site within 30 days of each other and report for the first time the genetic analysis of melanoma susceptibility genes in both twins. Data on characteristics of the twins were collected through a standardized questionnaire and skin examination. Exons 1α, 1β, 2 and 3 of *CDKN2A*, exon 2 of *CDK4*, the entire open reading frame of *MC1R* and the recently described *MITF* c.952 G > A (p.Glu318Lys) variant were investigated by direct sequencing. Sequencing analysis of the high-penetrance susceptibility genes showed no changes in *CDKN2A* and in exon 2 of the *CDK4* gene. Both patients were heterozygous for the same *CDKN2A* UTR c.*29C > G variant. Interestingly, the same two heterozygous variants of the *MC1R* were identified in both twins: the c.451C > T (p.Arg151Cys) and the c.456C > A (p.Tyr152*) variants. Neither patient showed the c.952 G > A (p.Glu318Lys) substitution in the *MITF* gene.

**Conclusions:**

Identification of two high-risk *MC1R* variants in our identical twins in the absence of *CDKN2A* and *CDK4* mutations highlights the contribution of low penetrance genes, such as *MC1R*, in melanoma susceptibility.

## Background

Genetic, phenotypic and environmental factors are known to affect melanoma risk. Two high-penetrance susceptibility genes, *CDKN2A* (cyclin-dependent kinase inhibitor 2A, MIM 600160) and *CDK4* (cyclin-dependent kinase 4, MIM 123829*)* and one low-penetrance gene, *MC1R* (melanocortin-1 receptor, MIM 155555), have been identified so far as genetic risk factors for melanoma [1-3]. *CDKN2A* is the major melanoma susceptibility gene, mutated in approximately 40 % of melanoma-prone families with at least three affected members. Predisposing mutations in *CDK4* have been identified in a limited number of melanoma families worldwide. Allelic variants in the *MC1R* gene, important in the pigmentation process, have been associated with increased melanoma risk, with the strongest effect observed for red hair color (RHC) variants (p.ArgR151Cys, p.Arg160Trp, p.Asp294His). Recently, whole genome sequencing lead to the identification of *MITF* (microphtalmia-associated transcription factor, MIM 156845) as a novel melanoma-predisposing gene with the p.Glu318Lys variant defined as an intermediate risk variant [4].

We describe the extraordinary occurrence of a primary cutaneous invasive melanoma in two 44-year-old female, identical twins, on the same body site within 30 days of each other and report for the first time genetic analysis of melanoma susceptibility genes in both twins.

## Case presentation

**Figure 1:**
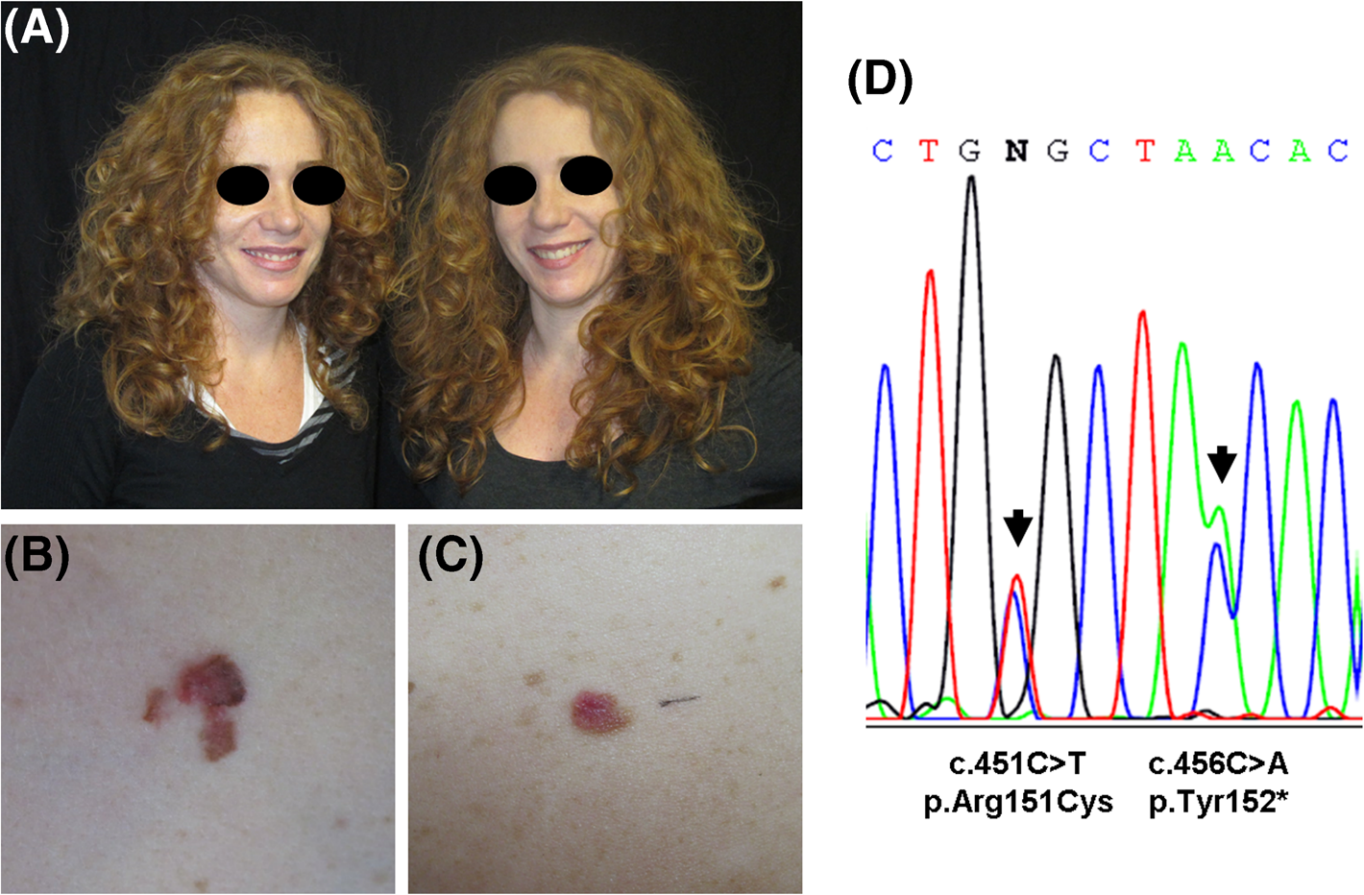
** Phenotypic appearance of the twins and of their melanomas and identified germline* MC1R* variants.** (**A**) Phenotypic features of twin-1 (left) and twin-2 (right); (**B**) Clinical image of twin-1’s melanoma located on the right anterior upper trunk; (**C**) Clinical image of twin-2’s melanoma excised from the right anterior lower trunk; (**D**) Heterozygous substitutions at codons 151 and 152 of the *MC1R* gene (NCBI accession NM_002386.3) identified in both twins.

A pair of female monozygotic twins who were included in a surveillance program at our Pigmented Lesion Clinic for the presence of multiple atypical nevi and at risk phenotype (Figure 1A) had been lost of follow-up over the last two years due to the 2009 earthquake in L’Aquila. Twin-1 presented with a one-year history of an irregularly-shaped, pink, light to dark brown, slowly growing plaque with irregular borders located on the right anterior upper trunk (Figure 1B). Thirty days later, twin-2 was examined for an asymmetrical, pink to light brown melanocytic lesion which had developed approximately 4 months earlier on the right anterior lower trunk (Figure 1C). Both patients referred a pre- existing pigmented lesion which changed in color and size. Dermatoscopic analysis showed irregular dots/globules, irregular streaks and a polymorphic vascular pattern in both cases. Histopathological examination showed a superficial spreading melanoma (SSM), 0.65 mm in Breslow thickness in twin-1 and a 0.33 mm thick SSM in twin-2. Both patients were skin type 1 (red hair, green eyes, fair-skinned, many ephelides) and were affected by the atypical mole syndrome with a total of 508 melanocytic nevi in twin-1 and of 375 nevi in twin-2. They reported numerous episodes of sunburns during childhood/adolescence, but different acute sun exposure habits in the adult life with longer recreational sun exposure (6 hours/day/4 weeks/year) for twin-1 than twin-2 (2 hours/day/2 weeks/year). They had neither personal nor family history of melanoma and twin-1 had three basal cell carcinomas excised.

### Genetic analysis

Genomic DNA was isolated from whole blood of both patients using QIAamp® DNA Blood kit (Qiagen, Hilden, Germany). Exons 1α, 1β, 2 and 3, including the exon-intron boundaries of *CDKN2A* (NCBI accession NM_000077.3)*,* exon 2 of *CDK4* (NCBI accession NM_000075.3) and the entire open reading frame of *MC1R* (NCBI accession NM_002386.3) were amplified using PCR and directly sequenced on ABI Prism® 310 Genetic Analyzer (Applied Biosystems, Foster City, CA) as described previously [5,6]. The recently described *MITF* c.952 G > A (p.Glu318Lys) variant (NCBI accession NM_000248.3) was screened by direct sequencing of a PCR-amplified 589 amplicon using the following primers: forward, 5'-TACATTCCCTCTGGTATTGT-3'; reverse, 5'-AGCAGTTTGTGCGAATGCA-3'. Primers for PCR amplification were designed from the *MITF* genomic nucleotide sequence (NCBI accession NG_011631.1) and were the followings: forward, 5'-TACATTCCCTCTGGTATTGT-3'; reverse 5'-AGCAGTTTGTGCGAATGCA-3'. An amplicon of 589 bp was obtained by standard PCR using 1.25 U of AmpliTaq Gold® 360 (Applied Biosystems) in a 50-μl volume, containing the 1X reaction buffer provided by the manufacturer, 1.6 mM of MgCl_2_, 200 μM of each deoxynucleoside triphosphate, 0.2 μM of each primer and 100 ng genomic DNA template. Five per cent dimethyl sulfoxide was added to the reaction solution. PCR amplification conditions were as follows: 95°C for 7 min, 35 cycles of 94°C for 1 min, 52.2°C for 1 min, and 72°C for 1 min, followed by a final extension step at 72°C for 7 min. Written informed consent was obtained from our patients under an Institutional Review Board-approved protocol.

Sequencing analysis showed no changes in the coding region and in the 5’UTR region of *CDKN2A*, although both patients were heterozygous for the same UTR c.*29C > G variant. No mutations were detected in exon 2 of the *CDK4* gene. Interestingly, sequencing of the *MC1R* identified the same two heterozygous variants in both twins: the c.451C > T (p.Arg151Cys) and the c.456C > A (p.Tyr152*) variants (Figure 1D). Neither patient showed the c.1075 G > A (p.Glu318Lys) substitution in the *MITF* gene.

## Conclusions

The occurrence of melanoma concordant for site and age of onset in monozygotic twins has been reported in only two pairs but in neither of them genetic analysis was performed. St-Arneault *et al*. [7] described two identical male twins (from a set of triplets) who developed melanoma at age 53, at the same site and within a time frame of 2 months, while the fraternal triplet showed no evidence of tumour. More recently, Rao *et al.*[8] reported two 71-year-old, identical female twins, diagnosed with melanoma at the same time (within 10 days of each other) and location (right calf). Our patients were skin type 1 as the latter pair but they were younger than both pairs.

Studies in twin pairs are of great interest to estimate the relative effect of genetic and environmental influences on melanoma development. In a recent large population-based study of Australian twins, genetic influences were shown to be a significant source of variation in liability to melanoma with identical twins being more than four times more likely to be affected with melanoma if they had an affected co-twin than non-identical twins [9].

*MC1R* variants have been associated with a 1.2 to 2.4-fold increased risk of melanoma with an additive effect for multiple variants [10-12]. A double variation in the *MC1R* gene, the c.451C > T (p.Arg151Cys) and the c.456C > A (p.Tyr152*), was detected in our twins. The p.Arg151Cys has been shown to confer the highest melanoma risk with summary estimates ranging from 1.78 (95%CI 1.45–2.20) to 1.93 (95%CI 1.54–2.41) in two recent meta-analyses [10,11]. We previously reported a significant association of the p.Arg151Cys allele with melanoma risk in a population from central Italy, with an OR of 2.94 (95%CI, 1.04–8.31) [6]. In functional studies, the p.Arg151Cys mutant receptor showed a reduced cell surface expression with intracellular retention and a corresponding impairment in cAMP coupling [13]. The nonsense p.Tyr152* is a rare RHC variant associated with a severe functional impairment of the MC1R due to the lack of the last four transmembrane fragments [14,15]. Interestingly, *MC1R*-associated melanoma risk has been shown to increase with the number of variants in the genotype supporting the *MC1R*-associated susceptibility in our patients carrying two nonfunctional *MC1R* variants. Finally, carriers of *MC1R* RHC variants have been recently suggested to develop melanomas lacking significant pigmentation [16,17] as also observed in the hypomelanotic melanomas of our twins.

In addition to the well-defined association of *MC1R* with melanoma, other low-penetrance melanoma risk alleles have been described in genes related to pigmentation, nevus count, immune responses, DNA repair, metabolism and the vitamin D receptor [12]. In this regard, we cannot rule out the possible involvement of other low-penetrance melanoma susceptibility alleles in our twins since they were not genotyped in our patients.

The concordance for cutaneous melanoma as well as the congruence for its location and identity of the age of onset in monozygotic twins support the role of genetic rather than environmental factors in the development of melanoma. Detection of two high-risk *MC1R* variants in our identical twins in the absence of *CDKN2A* and *CDK4* mutations highlights the contribution of low penetrance genes, such as *MC1R*, in melanoma susceptibility, although additional known or as yet unknown genetic risk factors might be involved.

## Consent

Written informed consent was obtained from both patients for publication of this case report and any accompanying images. A copy of the written consent is available for review by the Editor-in-Chief of this journal.

## Abbreviations

*CDKN2A*: Cyclin-dependent kinase inhibitor 2A; *CDK4*: Cyclin-dependent kinase 4; *MC1R*: Melanocortin-1 receptor; *MITF*: Microphtalmia-associated transcription factor; RHC: Red hair color; SSM: Superficial spreading melanoma.

## Competing interests

The authors declare that they have no competing interests.

## Authors’ contributions

All authors have read and approved the final manuscript. CP carried out the molecular genetic studies, participated in the analysis and interpretation of data and drafted the manuscript; MCF was involved in conception and design of the study, supervised the genetic analysis and participated in the interpretation of data and drafting the manuscript; MS collected the clinical data and participated in drafting the manuscript; KP diagnosed the patients, was involved in conception and design of the study and revised the manuscript critically.

## Pre-publication history

The pre-publication history for this paper can be accessed here:

http://www.biomedcentral.com/1471-2350/13/81/prepub
